# Supplementary figures and images for: IVEN: A quantitative tool to describe 3D cell position and neighbourhood reveals architectural changes in FGF4-treated preimplantation embryos
Source: PLoS Biol. 2021 Jul 26;19(7):e3001345. doi: 10.1371/journal.pbio.3001345 (PMC8341705; doi:10.1371/journal.pbio.3001345)

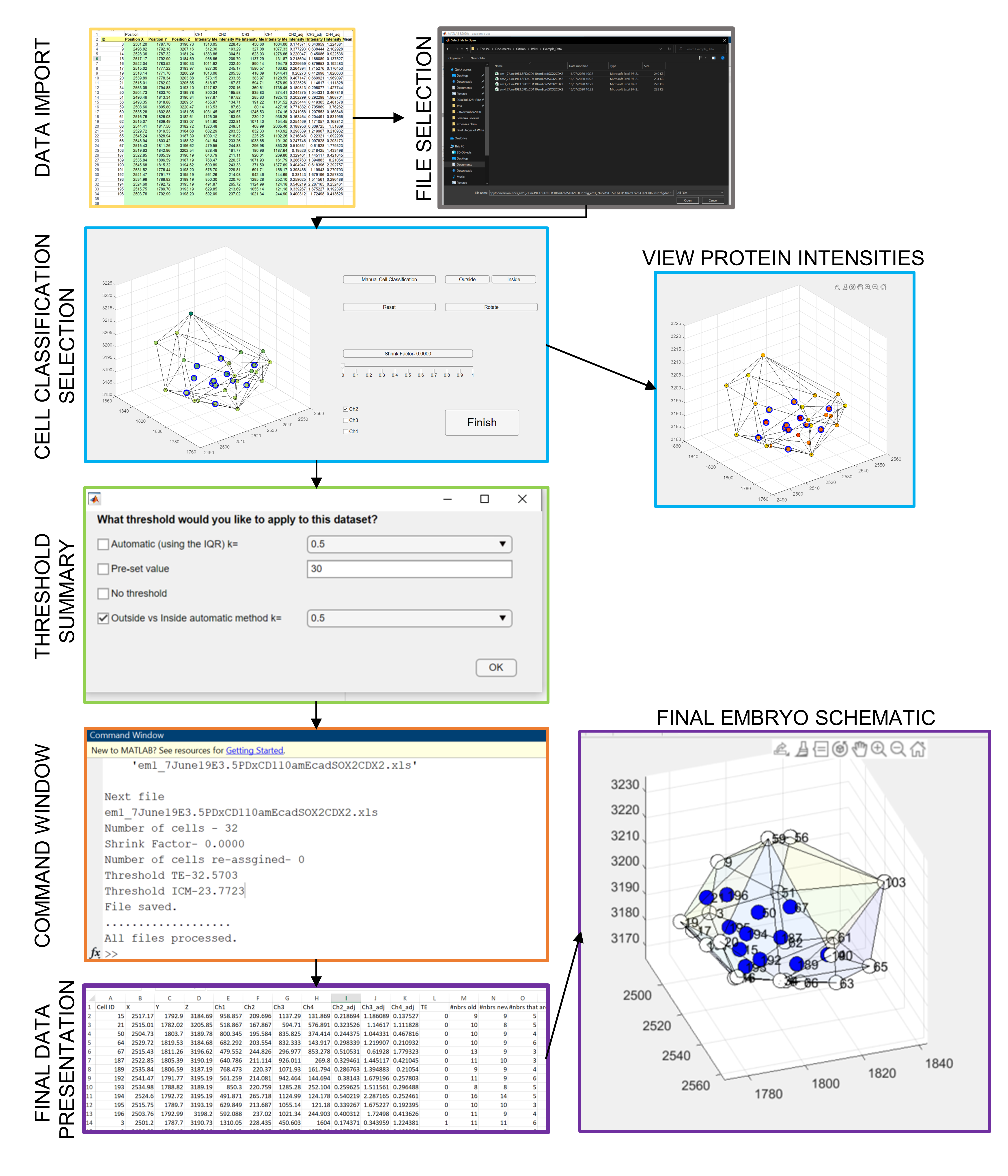

Supplement: S1 Fig — Data from IMARIS (or equivalent segmentation program) are output as an Excel file and compiled as outlined in tutorials in order to ensure that all data are imported correctly into IVEN (yellow box). Files are then selected using the standard file browser window (grey box). The embryo schematic and original cell classifications are generated and displayed to allow for user correction of the automatic cell classification (blue boxes). Channel intensities can be used to assist in correction of the cell classification if analysing confocal images. Method of thresholding of the DT is then chosen by the user, with a variety of options and tunable parameters (green box). After analysis, an output of thresholds used and number of cells analysed is output to the command window (orange box). Finally, an Excel file with all original input data as well as the numbers of neighbours and neighbourhood compositions is output, along with the embryo schematic with final cell classifications (purple boxes). DT, Delaunay triangulation; IVEN, Internal Versus External Neighbourhood. (TIF) [file pbio.3001345.s001.TIF]

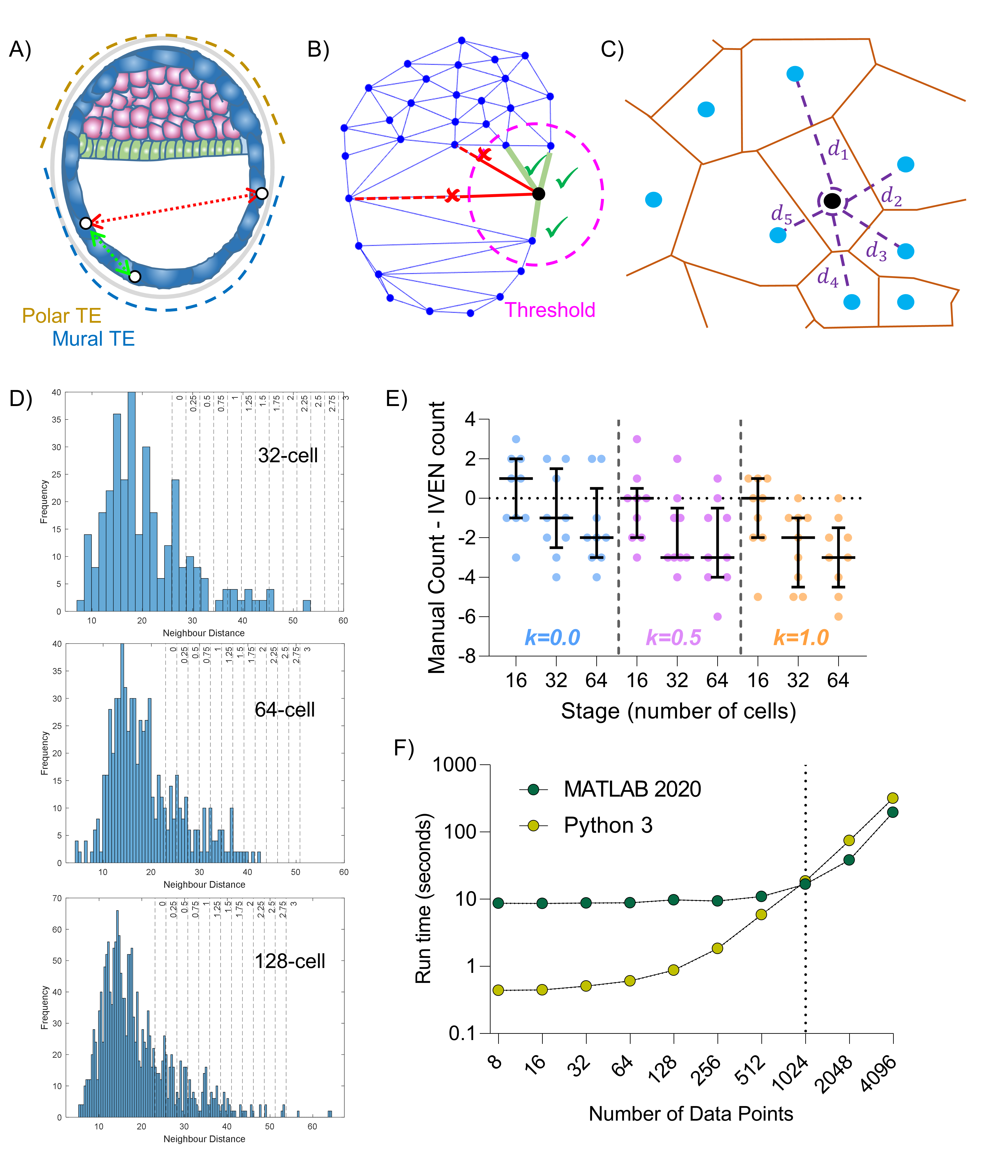

Supplement: S2 Fig — (A) Model embryo showing the effects of the cavity on mural TE cells and their neighbours. Untrue neighbours shown by red arrow, true neighbours shown by green line. (B) Two-dimensional in silico blastocyst with overlaid DT between nuclear centres (blue filled circles). Triangulation boundaries shown between opposing cavity cells (red lines) as well as true neighbours (green lines). Imposed threshold (pink circle) ensures that untrue matches are removed from further analysis. (C) Measurement of distances to all neighbouring cells in an in silico example. Orange lines show cell boundaries, blue circles represent cell/nuclei centres, and purple lines show Euclidean distances between neighbours. (D) Example distributions of distances between neighbours of cells within 32-cell, 64-cell, and 128-cell embryos. Dotted vertical lines show potential threshold boundaries as tuned through the value of k. (E) Manual testing of IVEN neighbourhood calculation using different values of k. Comparison of manual counts of neighbours with the neighbour counts as calculated using IVEN. (F) Approximate speed tests for the MATLAB and Python versions of IVEN. Approximate running times obtained by including generation of user interfaces with immediate closing of windows. Data underlying this figure can be found on the public GitHub repository https://github.com/jessforsyth/forsyth-et-al-2021. DT, Delaunay triangulation; IVEN, Internal Versus External Neighbourhood; TE, trophectoderm. (TIF) [file pbio.3001345.s002.TIF]

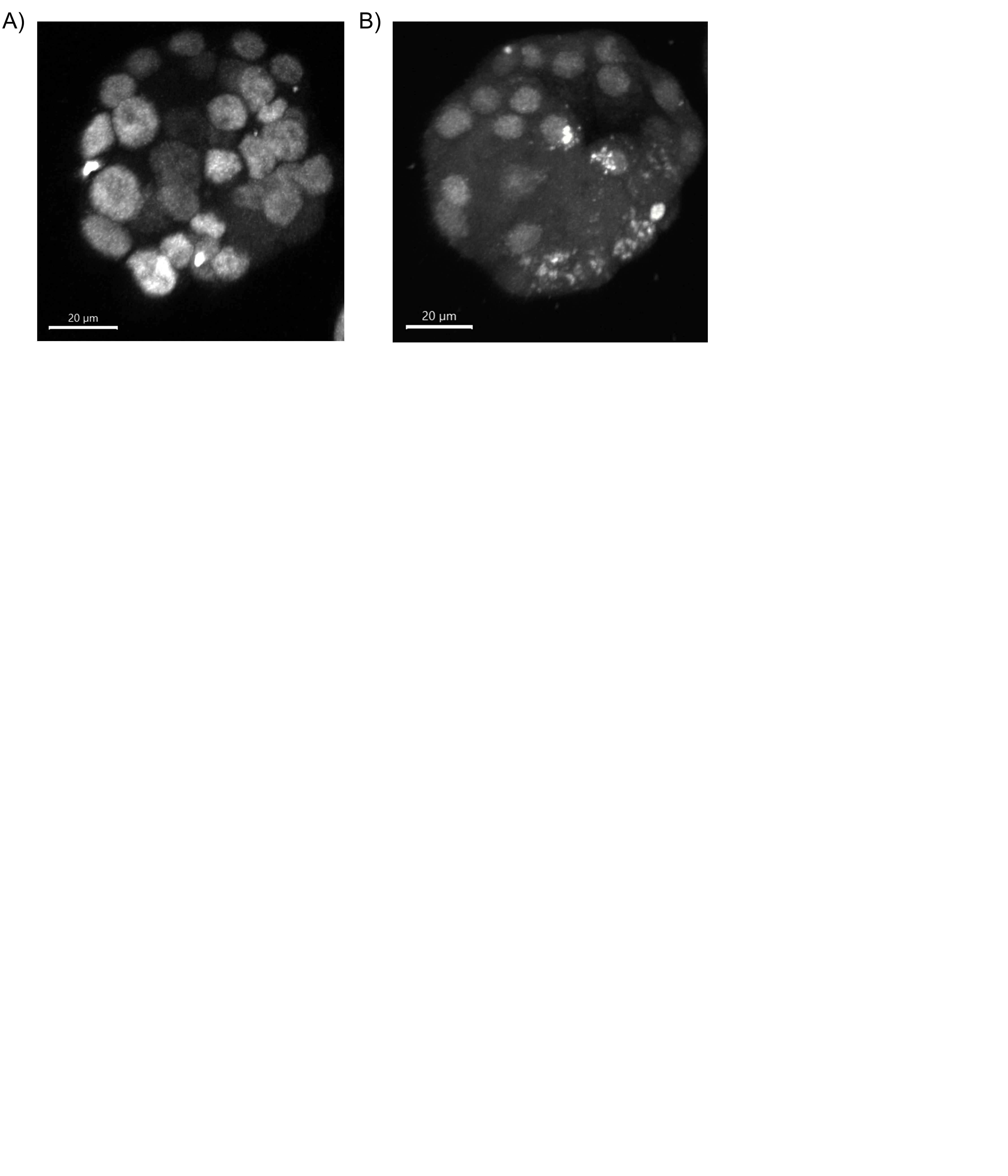

Supplement: S3 Fig — (A) Low background intensity staining at the 64-cell stage. (B) High background intensity staining at the 64-cell stage. (TIF) [file pbio.3001345.s003.TIF]

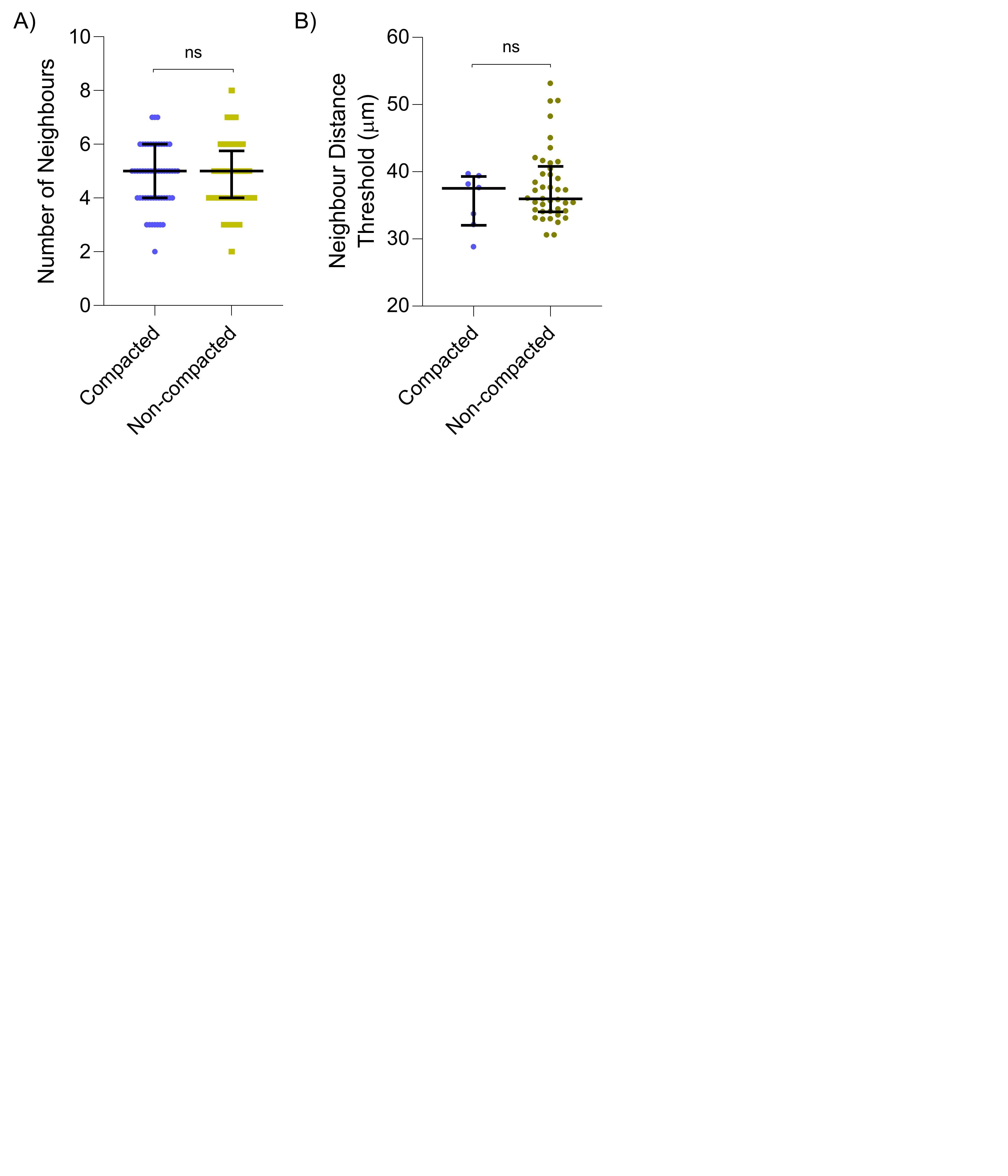

Supplement: S4 Fig — (A) Comparison of the number of neighbours of cells from 8-cell stage compacted and noncompacted morulae show no significant difference. (B) No evident difference between compacted morulae neighbour thresholds and noncompacted thresholds. Data underlying this figure can be found on the public GitHub repository https://github.com/jessforsyth/forsyth-et-al-2021. (TIF) [file pbio.3001345.s004.TIF]

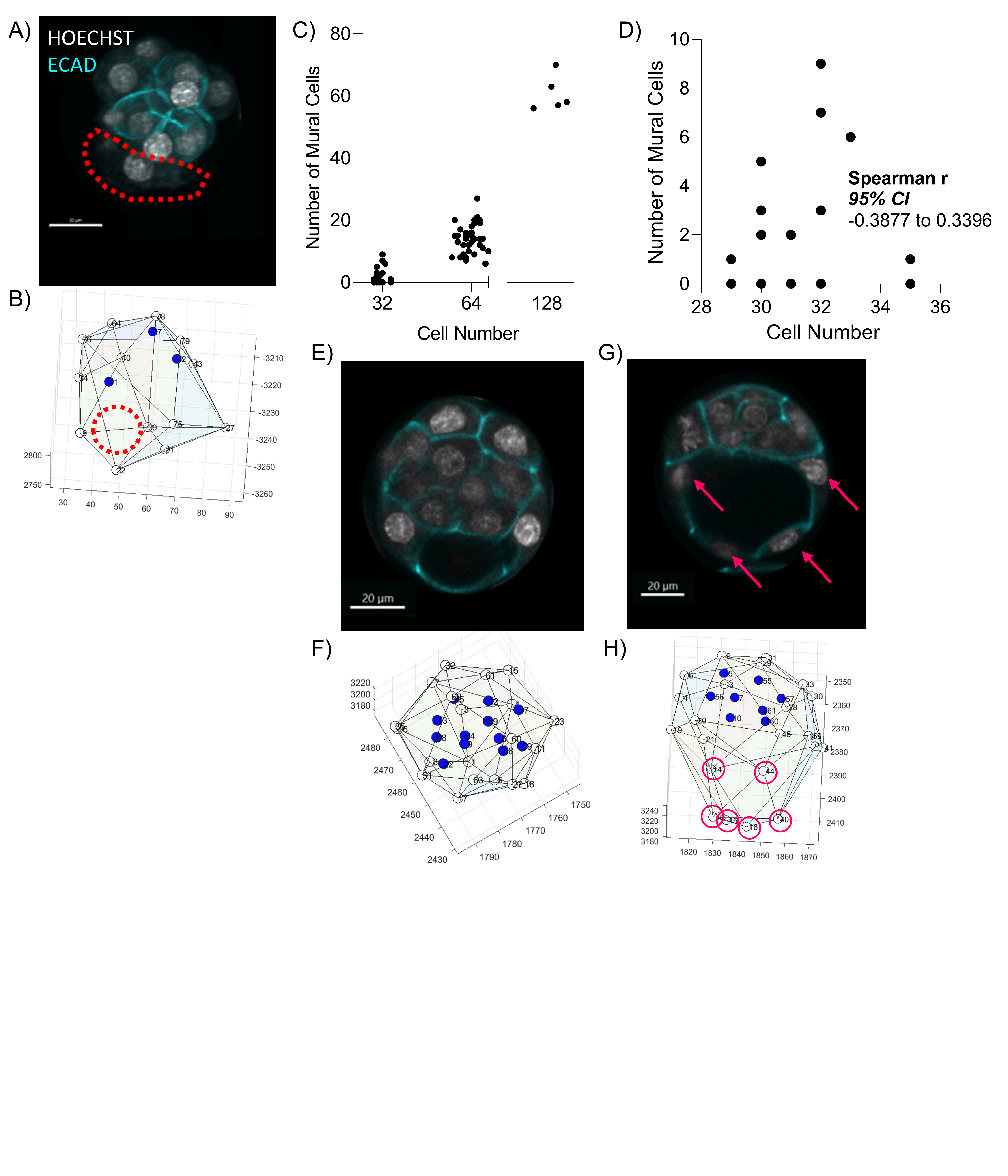

Supplement: S5 Fig — (A) Example 16-cell stage embryo with a cell (outlined in red) with only outside cells as neighbours. (B) Corresponding embryo schematic with highlighted cell. Slight elongation of morula captured in embryo schematic. (C) Increase in the number of identified mural TE cells against the size of the embryo (total number of cells). (D) Number of identified mural TE using IVEN against the total cell number at the 32-cell stage. No correlation evident between the number of cells around the 32-cell stage and the number of mural TE cells. (E) Example of a 32-cell stage embryo with a small cavity with no clear mural TE cells. (F) Corresponding embryo schematic showing no obvious cavity within the sample. (G) Example of a 32-cell stage with a more developed cavity and more obvious mural TE cells. Arrows indicate mural TE cells. (H) Corresponding embryo schematic with clearly visible cavity and mural cells indicated. Data underlying this figure can be found on the public GitHub repository https://github.com/jessforsyth/forsyth-et-al-2021. IVEN, Internal Versus External Neighbourhood; TE, trophectoderm. (TIF) [file pbio.3001345.s005.tif]

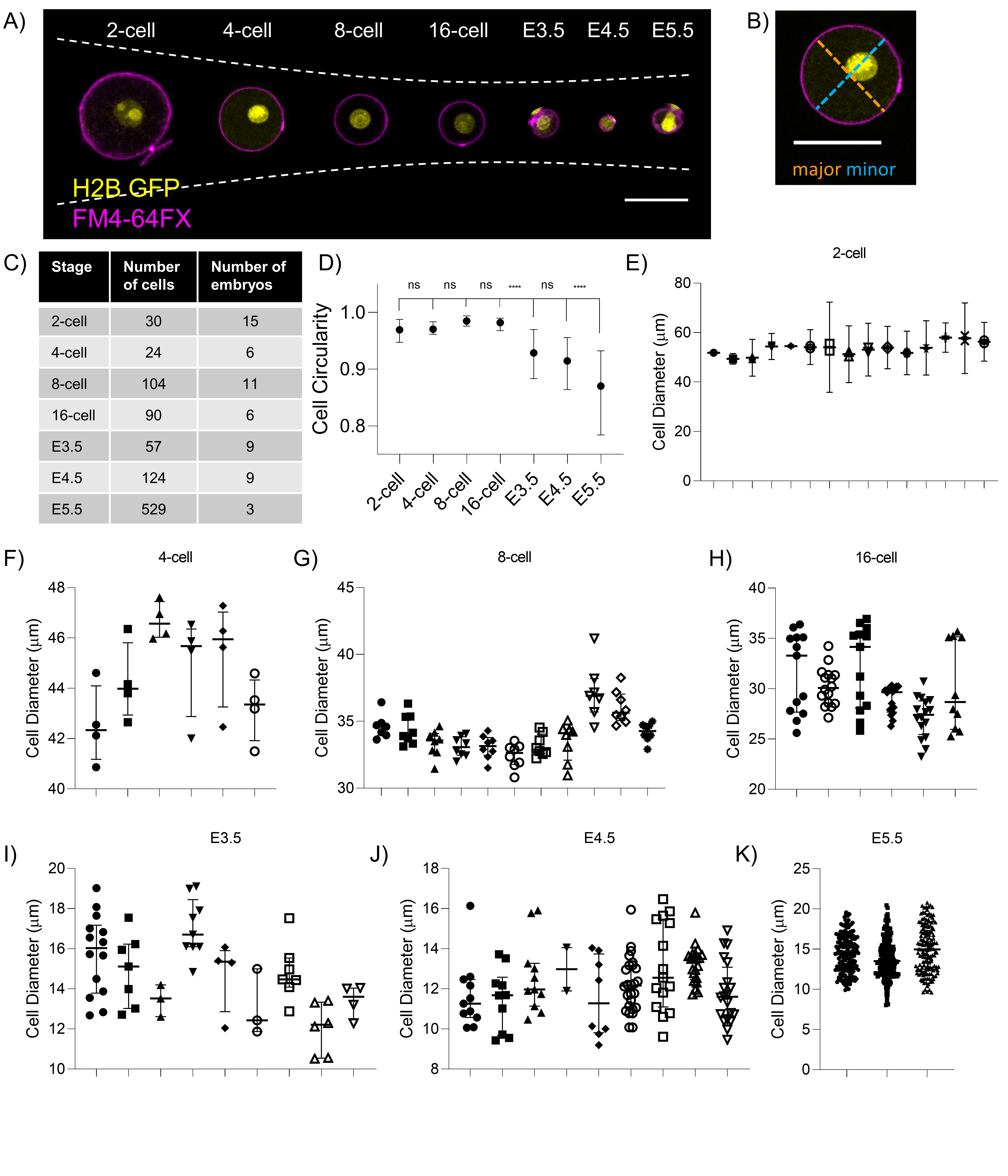

Supplement: S6 Fig — (A) Disaggregated cells from developmental stages prior to and around implantation. FM464-X dye used to mark cell membrane, H2B-GFP reporter line used to visualise nuclei. Scale bar, 40 μm. (B) Approach to measure the major and minor axes of disaggregated cells and their nuclei. Scale bar, 40 μm. (C) Cell and embryo numbers analysed. (D) Cell circularity close to one for all stages tested, supporting the assumption of the rounding of cells post-disaggregation. (E–K) Measurements of cell diameter for each embryo within each stage. Data underlying this figure can be found on the public GitHub repository https://github.com/jessforsyth/forsyth-et-al-2021. (TIF) [file pbio.3001345.s006.TIF]
