# Supplementary material for: IVEN: A quantitative tool to describe 3D cell position and neighbourhood reveals architectural changes in FGF4-treated preimplantation embryos
Source: PLoS Biol. 2021 Jul 26;19(7):e3001345. doi: 10.1371/journal.pbio.3001345 (PMC8341705; doi:10.1371/journal.pbio.3001345)
Supplement: Attachment — Submitted filename: JForsyth et al Response to Reviewers.docx [file pbio.3001345.s012.docx]

**Response to Reviewers**

**Forsyth et al. Manuscript June 2021**

Dear Editor,

Thank you for the opportunity to present the revised version of the manuscript ‘IVEN: A quantitative tool to describe 3D cell position and neighbourhood reveals architectural changes in FGF4 treated preimplantation embryos’.

In response to the comments by the editor, we have included an ethics statement in our materials and methods section regarding animal use within the project. This was provided by the animal facility at the University of Manchester where work was conducted. We have also reviewed the funding information for A.H.A and added the reference number that was stated in correspondence with Iraqi embassy. We cannot however provide any link to the program as this was not provided by the embassy. Therefore, the current summary of funding is as complete as is possible. We have also drafted a blurb ready for if our submission is successful. Additionally, we have uploaded data required for figures to the public GitHub and apologise for the delay. We include analysed excel files produced by IVEN where appropriate, and additionally present the Prism files that contain the data required to generate the figures and perform the statistical tests. If anything else is required, we would be happy for you to advise us.

We present the revised manuscript and figures in response to the four reviewer comments and thank all reviewers for their valuable comments and careful review of the manuscript. We summarise the major changes made to the manuscript here.

According to the suggestion of both reviewers 1 and 3, we have considerably rearranged and cut down the manuscript through removal of the nucleus diameter work (originally presented in Figure 5). We however elected to retain the cell diameter work as this work supports the phenomenon detected by IVEN of a decreasing neighbour distance threshold (and distance between neighbours, explained later).The remaining cell diameter work we have moved to Figure 2H alongside analysis of cell ‘spacing’ and thresholds within the embryo. Further to this, we have moved the original Figures 5A-C to supplementary Figure 5 in order to cut down the manuscript, and retain focus on the biological validation of IVEN results.

In conjunction with the above, it was suggested by reviewers 1 and 3, that the analysis and focus on the neighbour distance threshold was perhaps not as informative as it could be and that an assessment on the distance between cells and their neighbours would be more useful. We therefore implemented this analysis into IVEN and subsequently compared the median distance between cells and their neighbours for each developmental stage in Figure 2G. We now believe that this work presented in Figures 2F-H, show clearly how an initial observation in IVEN parameters have been analysed in more detail and then biologically validated through assessment of the cell diameter. We implemented the calculation of the median cell to neighbour distance in both the MATLAB and Python versions of IVEN as a default output value as we believe this to be an informative metric and good descriptor of biological architecture.

To further improve the default metrics output by IVEN, we also implemented the default output of the list of neighbouring cell IDs for each cell. We believe this information will assist in more detailed analyses of cell neighbourhood, and could be especially useful in further studies looking at protein expression at the single cell level. To make the IVEN pipeline useful for larger datasets, we also developed a batch processing version of the pipeline (in MATLAB) upon suggestion of reviewer 3.

Upon suggestion of reviewers 1 and 3, we also include a more thorough analysis of an alternative cell classification approach, namely ellipsoid fitting that has been used in previous cell classification pipelines. We include the comparison of the convex hull approach and a simplistic ellipsoid fitting in Figure 1E, and subsequently conclude that the convex hull is most suited for the IVEN pipeline due to its accuracy, flexibility and simplicity.

In response to reviewer 2’s questions regarding the presented CDX2 intensity values in Figure 1F, we realised that we had not fully explained our method of protein expression intensity normalisation and correction and have since included this explanation in the MM section of the manuscript. We also reviewed the datasets used within the figure and identified some embryos that had been analysed which had higher background intensity values than others, leading to increased CDX2 intensity values at the 64-cell stage in the inside cell population. We present a Response to Reviewer Figure to demonstrate the effect of this data with increased background but are pleased to present that even with non-perfect data, IVEN appears to be able to successfully classify inside and outside cells.

To address the question raised by reviewer 2 regarding the effect of compacted versus non-compacted 8-cell morulae, we manually classified all 8-cell stage embryos as either compacted or non-compacted morulae and identified 7 out of 48 embryos as compacted morulae. We then compared the calculated thresholds and numbers of neighbours for the two groups in Supplementary Figure 3. We identified no significant differences in the number of neighbours or threshold values and so group all 8-cell stage embryos together in analyses presented in Figure 3A-B.

In order to address the question raised by reviewer 1 regarding Figure 4E (now 4F) and the difference between neighbourhood distributions, we have increased the size of the plot in order to make it clearer for the reader and also have changed the plots to present the mean and SEM (as error bars) for all frequency distribution plots (upon suggestion of reviewer 3). We believe that the incorporation of error bars and increased size of the plots make the distributions more distinct and clearer to readers.

More generally, we have made an effort to reduce our ‘overanalysis’ of data that was highlighted by reviewer 3. This, in conjunction with streamlining of the manuscript and rearrangement of figures and relating text has reduced the overall length of the manuscript and we hope that this proves to be a positive change, making the manuscript more concise and informative. We have also taken care to arrange figures so that they are referenced in order to help the flow of the paper.

We address point-by-point (our answers presented in itallics) the individual major and minor comments of the reviewers below and include a Response to Reviewers figure for assistance in addressing point 6 by reviewer 2. Again, we would like to thank the editor for this opportunity and the reviewers for the comments that have undoubtedly improved the manuscript.

Best regards

Berenika Plusa

------------------

Reviewer #1: Review Forsyth et al. "IVEN: A quantitative tool to describe 3D cell position and neighbourhood reveals architectural changes in FGF4 treated preimplantation embryos"

Paper version April 2nd, 2021  - Review due April 23rd.

The spatial organization of cells in mammalian embryos can be very hard to grasp in 3D. This organization is however crucial, for the position of individual cells in an early embryo govern  their fate. Each cell integrates signals from its neighbors, and the presence, absence, numbers and identity of these neighbors is crucial to specify their lineage.
        In this article, Forsyth and colleagues introduce a new bioimage analysis tool called IVEN that helps investigate the identity of cells in a 3D embryonic sample, thanks to determining their neighbors. This software tool is distributed twice, as a MATLAB package and as a Python package. The latter has fewer features than the former, but can harness samples with a larger number of cells better. IVEN requires as input a CSV file that specifies the (X, Y, Z) position of all cells, which can be obtained via a 3rd-party tool. The boundary of the embryo is determined using the convex-hull, and IVEN allows for the manual correction of cells falsely labeled as inner cells by this algorithm. In a second step, IVEN determines cell neighbors by building a neighborhood graph using Delaunay triangulation. To prune aberrant edges that can be created in embryos with cavities (Figure 1C, green inset), IVEN automatically computes a distance threshold for cell-cell distance, above which edges
are deleted. IVEN then exports a new CSV file, with the class (inside vs outside) of a cell, the number of its neighbors, and the class of these neighbors. IVEN also uses MATLAB or Python libraries to offer 3D visualization of cell positions.  The authors then measure the accuracy of IVEN and illustrate its capabilities for mammalian preimplantation embryos studies. In particular, they study the neighborhood composition in mammalian embryos, and how it is altered in the presence of FGF4. They then measure cell and nuclei diameter along development. 

Although very long, the article is well written even for non embryologists. The software and its code are distributed on a public repository, which includes both the MATLAB and Python version. It also ships example data and excellent tutorials. The code is well written, and well commented. The program is split into several functions, again well documented. IVEN is really meant to be used by other scientists and the authors put a lot of effort into documentation and tutorials. I could run both the MATLAB and Python software successfully, albeit with minor difficulties when installing the required dependencies for the Python implementation (as always with academic Python code…)
 The analysis techniques used in IVEN are similar to e.g. the techniques used in Fischer et al., 2020 [22]. The authors of [22] also used Delaunay triangulation to build a neighborhood graph, with a distance threshold. But as noted in this work, a usable tool widely available was lacking so far. The novelty introduced by IVEN are mainly 1) a classification of inside vs outside cells based on convex-hull followed by manual correction and 2) an automated calculation of a distance threshold to prune aberrant edges in the neighborhood graph. As said above, IVEN keeps its promises and I have only a few major and minor points. 

*We thank the reviewer for taking the time to review the manuscript as well as testing the two versions of the IVEN platform and providing such insightful comments on the manuscript and how to advance IVEN and promote it to a wider audience. We address the major and minor comments outlined below.*


Major points.

1.
I feel like the last part (Cell diameter decreases with developmental stage until implantation & Nuclei change in size… & Figure 5) does not belong in the paper. It is a beautiful study well done and with meaningful results, but it does not rely on IVEN and does not illustrate its capabilities. Given that the article is already very long and is meant to demonstrate IVEN capabilities, I would suggest moving this part to another article.

*We thank the reviewer for this comment (along with Reviewer 3 who also made a similar comment). To address this point, we have removed the final section regarding the change in nucleus diameter due to its current indirect connection with the presented software (IVEN). We have however, retained the information regarding the change in cell diameter over the preimplantation period as this shows that the decrease in cell to neighbour distance can be associated with the biological phenomena of cell cleavage.*

2.
IVEN uses the convex-hull to classify cells as inside vs outside. The authors properly measure the performance of this technique (Figure 1E). In the discussion they state that they believe this approach is superior to the ellipsoid fitting approach used in MINS [14]. This point would be much stronger with an actual comparison of the two methods. For instance the study of Figure 1G could be made also with the ellipsoid fitting method.

*We thank the reviewer for this point and agree that some comparison of the two techniques/methods would add value to the study. In the revised manuscript we have addressed this point by using a pre-existing MATLAB ellipsoid fitting function and using the fitted ellipsoid to help identify the outside cells according to the protocol outlined below. We chose to use a pre-existing ellipsoid function to ensure fair comparison to the convex hull method due to its simplicity and ease of understanding within the code. Using the freely available function we identified the parameters of a suitably fitted ellipsoid and allocated a score (*$\nu$*) to each cell based on its coordinates*

$\nu=\left( \frac{x-o_{x}}{a} \right)^{2}+\left( \frac{y-o_{y}}{b} \right)^{2}+\left( \frac{z-o_{z}}{c} \right)^{2}$	*,*

*where x, y, and z were the coordinates of the cell,* $o_{x}, o_{y},$*and* $o_{z}$ *where the central coordinates of the fitted ellipsoid, and* $a, b,$ *and* $c$*the respective radii of the ellipsoid. If* $\nu>1$*then the point was allocated as an outside cell. This method is considerably simpler and more crude than that employed within MINS as we wanted to keep all methods incorporated within IVEN relatively simple so that even users not familiar with coding/programming would be able to interact with the software and modify it to suit their system. In the revised manuscript, we compare the convex hull algorithm with the ellipsoid fitting method and show the convex hull algorithm to be more accurate in the number of outside cells and so we implement the convex hull algorithm into IVEN as a method of cell classification.*

3.
In IVEN a distance threshold is automatically determined to prune aberrant edges, called in the paper neighbor distance threshold. It is calculated as P75 + 0.5 * IQR. In the third and fourth part (Figure 2G and 4B), the authors use this value as a measure of an embryo spatial organisation. Their conclusions are meaningful but my concern is that this threshold is an intermediate value used in a specific software (IVEN). For instance a researcher that wants to also measure this spatial organisation but without IVEN will have some difficulties relating their metrics to the metrics in Figures 2G and 4D. 
        I would like to suggest instead the use of a distance metric independent from IVEN process. For instance, the authors could define a mean distance to neighbors, defined for one cell as the mean distance from this cell to all its neighbors. Then plot the distribution of this metric for all the cells in one embryo, or take its median to compare from one embryo to another. This metric would carry the same information than the IVEN threshold but would be more readily generalizable.

*We agree that the maximum neighbour distance (as presented in the original version of the manuscript) may not be as familiar and easily interpretable to readers who do are not accustomed with the method. In the revised manuscript, we present the original threshold values in S2E Fig and instead show the calculated median neighbour distances in Fig 2F and 4C. We believe the incorporation of this metric makes the relation of the measurements/observed phenomena far more applicable to the observed decrease in cell diameter over the preimplantation period. In addition we added the calculation of the median neighbour distance to both the MATLAB and Python versions of IVEN so that this metric is output by default for every analysed cell.*

4.
In the same line, IVEN should offer more spatial features in its output. Right now it outputs for each cell the neighborhood composition, and allows incorporating fluorescence intensity. But it should also incorporate metrics on distance to neighbors, defined for instance as above, to ease compaction studies as is done in Figure 2G and 4D.

*As mentioned in the previous comment, we have incorporated the calculation of the median distance from a cell to its neighbours as a default output of IVEN (in both MATLAB and Python) and further to this we have included the output of a list of all cell neighbours (cell ID numbers) for each cell. We included this feature to enable better assessment of outliers and also to aid users in recreating the ‘networks’ of the environment. We also hope to encourage users of the platform to develop their own metrics for incorporation into the platform and hope to continue to develop the features of the software and the outputs available to users in the future.*

5.
Page 12, paragraph starting from line 458. The authors state that the differences in neighbor distribution in Figure 4E are evident, but I find it is not quite the case. The two distributions comparing control and FGF4-treated embryos have the same shape, the same mode. The differences are very subtle. For them to be relevant we would need to know whether we can trust the number of neighbor metrics with a precision better than 1 cell. Can IVEN reliably assess whether a cell has exactly 12 or 13 neighbors with such a high confidence? 
        This is not discussed in the paper and it is an important point. As stated by the authors, in the assays presented here we determine the number of neighbors via an indirect method (Delaunay triangulation) and not by directly observing contacts with membrane labelling.

*We thank the reviewer for this poignant comment that highlights several assumptions/points that underpin the interpretation of the results produced by the IVEN platform. To start with the initial query regarding the neighbourhood distributions presented in figure 4E (now 4F). Figure 4F now shows the two neighbourhood distributions (for KSOM and FGF4 treated) with associated error bars and show that the distribution for the FGF4 treated data is more peaked about 9/10 neighbours when compared to the KSOM distribution. Neither of the error bars overlap around this region, and we believe this to be a true phenomena of the experiment. Figure 4F has also been increased in size to make the differences in distributions clearer to readers.*

*As for whether IVEN can accurately describe whether a cell has* $x$ *neighbours or* $x+1$*neighbours, we employ the Delaunay Triangulation in the hope to avoid such subjectivity. For a given set of coordinates, there should be a unique triangulation that can be constructed (unless utilising a different method of constructing the triangulation). We use the DT as a mathematical and comparative description of the biological environment and so therefore describe the numbers of neighbours as robust values as output by the DT.*

Minor points.

6.      
Page 15 line 607. "Completely open-access". Your code is already open-access. Do you mean "without requiring the purchase of a MATLAB license"? 

*We thank the reviewer for highlighting this point, we have amended the manuscript to avoid confusion.*


Reviewer #2: The manuscript of Forsyth et al., describes the development of a new open source script (compatible for both Matlab and Python) called Internal Versus External Neighbourhood (IVEN) that permits automated classification of the position of individual cells (i.e. on the surface or inside) and their number of neighbouring cells (including distance metrics) within derived 3D microscopic datasets of imaged tissues (predicated on nuclear staining); thus, deriving important cellular architectural/structural information of tissue make up, under native or experimentally perturbed/pathological conditions. Importantly, the script also allows for user interaction to correct any (only limited) erroneous measurements and modulation of defined and tissue relevant parameters. 

The authors, use the preimplantation stages of mouse embryo development (in which they are extremely well versed) as a successful proof of IVEN concept. They show IVEN is able to reliably identify emergent outer and inner cells (based on the number of identified neighbouring cells any given cell, at a given developmental stage, has and thresholding the distance between such neighbours) and sub-categorise trophectoderm (TE) between mural- and polar-TE populations. Moreover, they are able to (for the first time and in exacting detail) conveniently and reliably identify the number of neighbouring cells in emerging ICM, mural- and polar TE cells and gain insight into the tissue architecture of these distinct blastocyst cell lineages. Their IVEN derived analysis also acted as a primer to identifying (again for the first time - in this reviewer's experience) the exact developmental stage reductive (in terms of cell size) cleavage divisions, typical of preimplantation developmental
stages, transit to more somatic like divisions preceded by cell growth (i.e. at the E4.5 peri-implantation stage). The authors also revealed the step-wise reduction in nuclear volume throughout preimplantation mouse embryo development. Lastly, the authors demonstrated how IVEN was able to detect and report modulation of blastocyst tissue architecture/structure (within the ICM) in response to exogenous FGF4 addition (known to promote a pan-ICM conversion to differentiating primitive endoderm/PrE cell fate at the expense of the pluripotent epiblast/EPI) that was consistent with transit towards formation of a 'double layer' of TE and PrE epithelia. 

Hence, this reviewer can see how IVEN is especially applicable to studies of preimplantation mouse (and mammalian embryos) and would be applicable to the reanalysis if many existing data sets (including pharmacological inhibitor treatments, clonal RNA-mediated specific gene expression knockdowns or genetic knockout models affecting cell fate or the derivation of the hatching blastocyst). Moreover, as the authors suggest, IVEN is equally applicable to other examples of the study of tissue morphogenesis (e.g. organoids, gastruloids, small tumours etc.) 

In compiling this review, the reviewer acknowledges they were assisted by a senior researcher in their lab (particularly given the manuscript is submitted as a "Methods and Resources Article"). This reviewer found the manuscript highly original and innovative. It is well written, easily followed and will be of interest and utility to both researchers within the mammalian preimplantation developmental field and beyond. As indicated above, the described experiments not only provide a proof of the developed (and widely applicable) IVEN concept, they also provide novel insights into mouse blastocyst tissue morphogenesis. Therefore, this reviewer is quite happy to recommend publication of the manuscript in PLOS Biology but would welcome some redress to few specific points. 

*We thank the reviewer for their thorough review of the manuscript and their appreciation for IVEN’s place within the research field. We also thank the reviewer for their more probing biological questions. We address the specific comments raised below.*

Specific points:

1)      This reviewer is curious if the authors, after highlighting the "three fundamental embryo scale architectural changes occurring during the preimplantation period" (first line of the Results section - line 183), conducted IVEN analysis on both non-compacted and compacted 8-cell stage embryos? One asks because it could be expected that the comparison of 'neighbourhood composition' would not significantly change but the 'neighbourhood distance threshold' would reduce.

*We thank the reviewer for this interesting comment and suggestion. To address this question, we categorised each embryo as compacted or non-compacted by using the brightfield microscope images (as embryo schematics generated by IVEN showed no obvious features to help identify compaction). We identified 7 out of the 48 analysed embryos as compacted morulae and compared the number of neighbours of the cells between the two groups and the calculated neighbour distance thresholds, we present these comparisons in Supp Fig 3 to make this observation clear to the reader. We identified no statistical differences between either comparisons, and hypothesise this to be due to the high cell to nucleus volume ratio at the 8-cell stage. As IVEN uses only the nuclei centre coordinates to model the embryo, we hypothesise that the nuclei in fact do not ‘move’/’shift’ significantly during compaction and that instead it is the surrounding cell that is predominantly affected during compaction.*

2)      This reviewer is also curious about the observation that 16-cell stage nuclei (in individual cells derived from disaggregated embryos) exhibit a strong degree of sphericalness but the blastocyst stages show much more heterogeneity with substantial populations of ellipsoid nuclei (particularly by E4.5). Do the authors consider the formation of the fluid filled cavity (and potential exertion of mechanical forces, particularly in the epithelial mural-TE and later PrE) a contributing factor? How does the relative distribution of such sphericalness correlate with the IVEN identification of mural-TE (if it is possible to measure nuclear sphericalness in intact blastocysts reliably)? This reviewer also notes from the authors' Discussion section, the observed blastocyst stage increases in the variation of nuclei size and shape is speculated to be related to initiation/progression of cell differentiation/specification. Whilst one agrees this is highly likely, would it not have
been relatively easy to stain disaggregated cells for specific blastocyst cell lineage protein markers (e.g. CDX2 for TE or SOX17/GATA4 for PrE etc.) to directly test this? However, this is not necessarily a means to delay publication but would conversely strengthen the manuscript.

*We thank the reviewer for these brilliant points and interesting questions. However in the revised version of the manuscript, we followed the advice of reviewers 1 and 3 and have removed the quantification of nuclear diameter and sphericity in order to retain the focus of the manuscript on the IVEN pipeline and associated analyses. We would however like to address such questions, such as the relationship between nuclear morphology and cell specification, in another manuscript in the future and hope to assess these differences more comprehensively by, for instance, including some fate specific analysis of nuclei diameter. We hope that such analyses would potentially highlight some of the earliest differences in sub-cellular morphology and that such revelations would prove useful to the community.*

3)      Similarly, the authors suspect those derived inner cells that only have other neighbouring inner cells may be under "differential signalling regimes" (line 347) that could ultimately effect ICM cell fate. Could they not co-IF stain developing blastocysts for PrE/EPI marker proteins in order to categorise the (emerging) fate of this population?

*We thank the reviewer for this suggestion and hope to address this is future works. We feel that this analysis may be slightly too specific for this manuscript where we are primarily trying to introduce IVEN and its features, but we hope to address such questions in a more comprehensive future manuscript.*

4)      Relating to Fig.4: Can the authors provide evidence that their +FGF4 treatment regime did not (or indeed did) change overall cell numbers? As cell number changes could contribute to changes in tissue architecture that they observed and may merit further discussion.

*We thank the reviewers for this important point. We present the numbers of ICM cells as identified using IVEN (Fig 4B) and present no significant differences between the two treatment regimes. Though we do acknowledge that the number of ICM cells in FGF4 treated embryos is more variable, the overall median ICM number appears to be similar to that presented in KSOM cultured embryos.*

5)      Regarding the comparison of the authors data with that of Aiken et al., (line 509), this reviewer does not consider the statement fair/valid given one study focussed on cells within in intact embryos and the authors data refer to disaggregated spherical cells.

*We express thanks for the careful consideration of comparative studies and have amended the manuscript to highlight the difference in approach more clearly as to avoid confusion.*

6)      In relation to Fig. 1F (CDX2 intensity expression analysis in IVEN called inner and outer cell populations): Do the authors agree that inner CDX2 expression is equivalent at the 16-cell and 64-cell stages (as depicted in the figure)? Alternatively, do the reported data indicate IVEN is not so robust in reliably identifying inner and outer cell populations at this stage (and potentially beyond)?

Response to Reviewer Figure 1 A) Example of a 3D view of an embryo stained for CDX2 with background intensity present within the ICM. B) Example of a 3D view of an embryo stained for CDX2 with minimal background intensity present within the ICM. C) Original figure comparing the normalised CDX2 intensity between inside and outside cells, as classified by IVEN. D) Modified figure, with the removal of 64-cell stage embryos with significant background intensity present within the ICM. (n=3 embryos at the 64-cell stage).

*We thank the reviewer for this close inspection of Fig 1F. To address this point we manually reviewed the microscopy images of all CDX2 stainings. All images used showed outer cells to have noticeably increased levels of intensity, however some images were shown to have high levels of background (Response to Reviewer Figure 1A) compared with other images (Response to Reviewer Figure 1B). During the preparation of this manuscript and testing of IVEN we had to rely on data produced before pandemic, mainly created by undergraduate students. As result we decide to include both high quality data as well as high background data. We present the original figure (Response to Reviewer Figure 1C) with the removal of embryos with increased background (Response to Reviewer Figure 1D) for comparison.*

*With the removal of the data, the success in cell classification is more evident, however we are pleased to show the successful performance of the IVEN pipeline even when handling non-perfect data. We also believe that including data generated by 3^rd^ year undergraduate students, gives them important confidence boost, and while these data are not perfect but still illustrate efficiency of IVEN, despite noise in the data.*

*However, if the reviewer believes that for the sake of clarity we should remove “noisy” data, we could potentially replace figure 1F and only include low-noise data.*

*Additionally, thanks to the reviewer comments, we realised that we did not explain our methods of normalising data in sufficient detail. We have now rectified that by adding more extensive explanation of our normalisation method in MM section. In short, raw cell intensity values are first corrected for reduction in signal due to signal attenuation by division by the nuclear signal (Hoechst staining, assumed to be equal in all cells – i.e. any decay in signal is solely due to depth attenuation). Corrected cell intensities are subsequently normalised per embryo by division by the maximum cell intensity value for each channel. This method therefore leads to all intensity values ranging within 0 and 1. Therefore our method to normalise intensity values per embryo unfortunately does not always allow for meaningful comparisons of CDX2 intensity across stages. For instance a 16-cell stage CDX2 intensity of 0.5, may not correspond with the CDX2 intensity of 0.5 from a 64-cell stage embryo.*

7)      The legend to Fig. 3 lacks reference to panel E) describing neighbourhood frequency distributions at the E4.5/128-cell stage.

*We thank the reviewer for noting this point and hope that we have amended all erroneous/missing figure legends.*

Reviewer #3: Morphogenetic changes of embryos are driven by developmental processes at the cellular level including cell differentiation, division and rearrangements, which are tightly coordinated and ultimately determine the success of the embryo development. In the present work, Forsyth et al. introduce IVEN, an interactive pipeline to quantitatively describe changes in the environment of individual cells within growing mammalian embryos. In particular, IVEN discriminates between internal (inner cell mass) and external (trophectoderm) cells using neighbourhood analysis. The analysis is based on Delaunay triangulation, a classical triangulation method used to connect points in space generating a convex hull of the point set, and modified to remove point connections with a length higher than a threshold, either computed in an automated fashion or determined manually. The software is written in MatLab and Python, and clear tutorials are provided for both options, making it a
tool with a potential to be widely applicable.

The authors apply the approach to the study of the developing mouse embryo, in which a fluid-filled cavity is formed during trophectoderm maturation. The authors use the IVEN pipeline to classify cells in different clusters based on their relative positions, and further demonstrate that FGF4 treatment causes changes in the organization and distribution of cells within the embryo. Finally, the authors show that the diameter of cells and nuclei is decreasing between the 2-cell stage and E4.5, after which they observe a subtle increase in diameter. This work provides an easy-to-follow pipeline to perform a systematic analysis of cell distribution within embryos. The article is clearly written and the method is well explained. However there are some issues that need to be addressed and we will recommend publication upon addressing these concerns.

*We thank this reviewer for taking the time to review the manuscript and provide such perceptive comments. We address each comment individually below, some of which were also raised and addressed in the comments provided by Reviewer 1.*

Major Concerns 
1. In general the text is very long with unnecessary descriptions and inferences from over analysis of data. 20 pages of text with 5 main figures seem like an overkill. Figure 2D can be seen as an example where the green box shows that inside cells exhibit a sub-population of cells with 100% of their neighbours also inside cells. I am not sure what it means to report values like 0.3%, 1.9% and 2.3% when dealing with stages that consist of such few cells (32, 64 and 128 cells respectively). This is one instance where an "overanalysis" adds to the length of the text without necessarily offering a conclusion that provides any deep biological insights. The work is very interesting and the technical achievement is quite adequate to deserve publication as a crisp and to-the-point text without the need for overselling through long text.
*We express thanks to the reviewer for this comment and have made efforts to reduce the length of the text and remove some of the sections of ‘overanalysis’ such as the quantification of inside cell composition subpopulations. As mentioned in the response to Reviewer 1’s comments, we also elected to remove the work on nuclear diameter due to the lack of a clear, direct link to the overall embryo architecture at this time and hope that this works towards successfully condensing the manuscript.*

2. Along the lines of comment above, I fail to grasp the point of section starting at line 393. Why using the distance threshold as a proxy of cell packing, when cell positions are available and therefore the density and average distance of cells can be directly computed after IVEN cell classification? A similar question comes for the next section, where cell dispersion in FGF4 treated embryos can be directly computed with cell position and classification.

*Again we thank the reviewer for this comment (in conjunction with the comment from Reviewer 1). We have since implemented a default output of IVEN of the median distance from a cell to its neighbours as a means to more clearly explore the idea of cell packing and the observed phenomena of the decreasing neighbour distance thresholds and present this data in figures 2F and 4C. We also hope that this additional parameter will help link the outputs from IVEN with other existing studies that explore cell density and/or packing.*

3. It seems that the two last sections on cell dimensions are disconnected from the rest of the text and from the description of the software. While they provide interesting observations, they fail to integrate within the technical framework of the paper. This connects to point 7: why putting so much effort in justifying a meaning for the threshold parameter?

*We thank the reviewer for this comment and advice on how to condense the paper for the readers. In the revised manuscript, we have removed the section regarding the changes in nucleus diameter over the preimplantation period but have opted to retain the assessment of the cell diameter, as we hope this provides a biological exploration of some of the phenomena detected by IVEN such as the reduction in the distance between cells and their neighbours with developmental stage. In doing so, we also show, to our knowledge, for the first time, the point at which cells begin to increase in size within mammalian development.*

Minor concerns 

1. While the pipeline is clear and easy to follow and the manual inspection of the data gives high flexibility in choosing the right Delaunay threshold, the time required to process large amounts of data can quickly increase (as shown in Sup Fig 2F). A batch processing module capable of processing several files within the same folder using default parameters would greatly improve the applicability of IVEN to e.g. large time lapse datasets. The final results could still be manually inspected with the standard IVEN pipeline to correct classification errors.

*We thank the reviewer for this fantastic suggestion and have developed and provided a batch process version of the IVEN script in MATLAB on the public GitHub repository. We appreciate that the analysis of large datasets can be time consuming, however we will continue to suggest that users default to use the original IVEN version (complete with secondary manual analysis of cell classification via the GUIs) to ensure accurate cell classification. For more simple systems or systems with more regular shapes, we believe the batch process method will prove to be accurate and time-effective.*

2. Fig.1: how are the cells labeled in panel F: are those cells based on IVEN classification? If so, why are there some inner cells with high cdx2 expression and some outer cells with low cdx2 expression? Are these the cells that are mislabeled as in panel E? What is "ne" in panel E? Most likely it is the number of embryos analyzed and that should be mentioned in the figure legend.

*We thank the reviewer for highlighting this confusion, the figure does present cell classification as performed by IVEN and have made an effort to make this clearer in the text and figure legend for figure 1F. As described in our response to Reviewer 2, the discrepancies in CDX2 expression at the 64-cell stage were largely effects of high background noise within images used during analysis. We chose to use images generated by undergraduate students during the pandemic to increase sample sizes. We still demonstrate a significant difference in CDX2 expression between inside and outside groups at the 64-cell stage despite the high background level and so believe this to be a successful performance of the convex hull. We however appreciate if the reviewers would advise to remove the data with high background levels in order to make the successful performance of the convex hull clearer to readers.*

3. Why is convex hull a better way to determine outside cells when compared to ellipsoid fitting? Both methods are not capable of describing complex shapes (as explained in lines 206-210). Is the power of IVEN in the manual curation of the datasets? A discussion on this point would help the reader in understanding the advantages of IVEN over ellipsoid fitting.

*We thank the reviewer for this question and address this point by comparing a simplistic ellipsoid fitting technique with the convex hull approach in figure 1E. We show that the convex hull method of classifying outside cells is superior to basic ellipsoid fitting techniques, at least in the context of the preimplantation embryo. We do however acknowledge that in conjunction with more sophisticated fitting techniques, such as those employed in MINS (Lou 2014), the ellipsoid fitting approach may be appropriate for some systems and analyses. We however wanted to use a simple methods to classify cells so that even users not familiar with coding/programming would be able to interact with the software and modify it to suit their system most appropriately. We therefore surmise (and present) that the convex hull method is most accurate when comparing to a simplistic ellipsoid fitting approach.*

*We agree with the reviewer that a large asset of IVEN is the ability to manually curate cell classification using the GUI, but also in the very nature of the convex hull algorithm that will likely be more applicable to a wider variety of biological systems such as tumours, organoids etc.*

4. Missing legend for sup fig 2F

*We thank the reviewer for finding this and hope we have made amends to this mistake in the figure legends.*

5. Consider moving Fig 3 as Sup Fig, as the main text now jumps from fig2F to fig 3 and back to fig2G.

*We agree with the reviewer that this caused the manuscript to feel like it was jumping between sections and rather than move Fig 3 to the supplementary material, we have decided to incorporate Fig 2G (now Fig 3A) into Figure 3 as to help the reader further explore the neighbourhood changes over the preimplantation period within the same figure. We also hope to offer these two methods of neighbourhood analysis (absolute number versus frequency distribution) as valuable techniques to explore cell environment and neighbourhood and so have opted to keep both methods within the main figure document.*

6. General comment on the distribution plots: it would be better to show the average distribution among all embryos analyzed and the errorbar around the mean, rather than pulling all embryo data together in a single distribution. This would help understand, e.g. in Fig.4E, if the differences in the distribution of control and FGF4-treated embryos are significant.

*We thank the reviewer for this comment, we agree that by changing the plots to this format we are able to see the variability of the data (embryo to embryo) more clearly as well as assessing the strength of an observation. We therefore present the average frequency distribution along with an error bounded region that is representative of the standard error of the mean for all frequency distribution plots within the manuscript.*

7. All Supplementary figures are referred as Sx Fig instead of Fig Sx (example lines 195, 224, etc). This should be rectified.

*We thank the reviewer for help on the formatting of the figure references, we have checked with the submission guidelines and hope that our current format fits with the PLOS biology format outlined. We are happy to be advised otherwise and will make the necessary changes.*


Reviewer #4: This manuscript by Forsyth and colleagues describes the development of an open-source pipeline that can be used to position cells with respect to where they lie in the embryo as well as determine the number of neighbours that each cell has. The authors first validate the use of this pipeline by analysing pre-implantation mouse embryos and find that it accurately can distinguish inside from outside cells. They then use the pipeline to analyse how the neighbourhood of each of these cell populations changes during pre-implantation development as well as in response to FGF4 treatment, that induces cells to form a primitive endoderm fate. Importantly, they find that significant changes in number of neighbours and degree of cell packing correlates with when cell fates are being specified, suggesting that these changes may contribute to lineage decisions.  Finally, by analysing the changes in nuclear size that occurs during pre- and early post-implantation development
they determine that cell division reduces cell size until the post-implantation period when cell growth is restored. Overall, the manuscript reports a very useful tool to study the relationship between embryo geometry and cell neighbourhood and the authors use it to uncover important new principles governing early mammalian development. 

There are just some minor points that would strengthen the manuscript if they could be addressed.

*We thank the reviewer for reading the manuscript and comments. With regards to the questions raised, though we do not address these comments in the manuscript, we thank the reviewer for their insight into the potential applications of IVEN and potential questions that such analyses could lead to.*

1.      Can the system be used to detect dying cells? If so, what is their neighbourhood?

*We thank the reviewer for this probing question. With our current knowledge of cells and their neighbourhood we are not able to detect dying cells explicitly from their environment. However if dying cells were identified using an existing software e.g. IMARIS or Fiji, and their Cell ID noted, their environment could be assessed using the output excel files generated by IVEN. We have also added in the feature of outputting the cell IDs of all neighbours of cells and so cells neighbouring a dying cell can also be easily identified. If dying cells were found to have a specific feature of environment or neighbourhood, this could then be included into IVEN to predict whether a cell is dying or not. This would form a particularly interesting application of IVEN and hope to pursue this further in future works.*

2.      Can the authors comment on if changes in in cell neighbourhood and packing  precede when the different cell fate choices occur during pre-implantation development, or if they occur after lineage specification events and are therefore likely a consequence of these events?

*We thank the reviewer for this ‘big picture’ question. At this point in time, we cannot comment with any surety whether the change in environment drives lineage specification (or simply precedes it) or whether it takes place after lineage specification as a result of cell specific properties. We hope that with IVEN as a well-established tool to assess cell environment, that we will be able to assess this relationship and distinguish the nature of the environmental changes taking place within the preimplantation period through assessment of live imaging studies and intermediate stages. However, we are well aware that some signalling pathways respond to mechanical forces and so hypothesise that there is some relationship between cell fate and cell neighbourhood, whether it directs cell fate acquisition or helps re-inforce it.*
